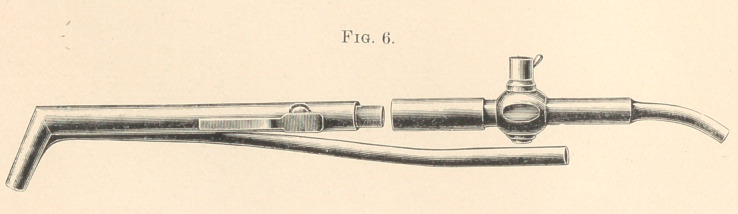# Crown- and Bridge-Work

**Published:** 1893-03

**Authors:** C. M. Richmond

**Affiliations:** New York


### International Dental Journal.

Vol. XIV. March, 1893. No. 3.

### Original Communications.[1]

### CROWN- AND BRIDGE-WORK.[2]

BY DR. C. M. RICHMOND, NEW YORK.

(Continued from page 86.)

In the treatment of badly-decayed bicuspids where the cavities extend from the mesial to the distal surface, leaving the cusps standing and in good condition, I separate with disk to the gum-line and thoroughly excavate. If the pulp be complicated in the case, I remove it and prepare as if for a filling. The next step in the operation is to fill the tooth with hard wax, so that a perfect impression can be taken in plaster for the purpose of getting a fusible metal die of the tooth. A gold band is now carefully fitted to the tooth, letting it extend to the gum-line entirely around the tooth; I cut out the front of the band which covers the facial surface of the tooth so that only a narrow band shows, leaving the band where it passes between the teeth fully up to the cutting-edge of the tooth. The cavity is now dried out, and also the outer surfaces, cement mixed, and the tooth filled with sufficient to leave a surplus, placing some of the cement on the outside surfaces. The band is now thoroughly covered with the cement on the

[1] The editor and publishers are not responsible for the views of authors of papers published in this department, nor for any claim to novelty, or otherwise, that may be made by them. No papers will be received for this department that have appeared in any other journal published in the country.

[2] Copyrighted, 1893, by Dr. C. M. Richmond.

inside surface, and placed on the tooth and carried home. After the cement has crystallized the surplus is removed, and we have a tooth that is perfectly protected from any further destructive process.

To further make this operation durable, I place the rubber dam over the teeth and cut out enough of the cement to give anchorage for a gold filling. I now fill from side to side against the gold band, and, when finished, polish as in an ordinary gold filling. This operation is easily performed in one sitting, and where the patient objects to a whole gold crown, or to having the tooth cut off for a crown of porcelain, this furnishes an easy way out of a difficulty.

In Fig. 1 I have illustrated a half-crown for restoring a bicuspid where the buccal surface or cusp has been lost. This crown is shown without a pin or post, as it would be made for a tooth where the nerve is still alive and it is desired to keep it so; the pin or post would be used in combination with the half-band if the pulp had been lost. In making this operation I bevel the broken surface to the gum-line and shape the standing cusp as desired. A plaster impression is then taken of the tooth, as before described. After casting a die of metal, a band is bent and fitted, letting the ends lap where it covers the broken surface of the standing cusp; I solder and fit the half-band as perfectly as if for a whole crown. After fitting it on the standing cusp in the mouth, a small eye-tooth is selected and ground to a bicuspid shape, and the bevel ground to fit the bevelled surface which has been made in grinding root in the mouth. The case is now waxed together and tried on in the mouth; if right, it is invested, and a small piece of solder is all that is required to secure the porcelain to the band where it is lapped and soldered. If the tooth has lost the pulp, I always use a post in combination with the band, thereby securing every advantage to be gained by the combination of band and post. After the case is ready to fasten in position I dry the root thoroughly, and also the band, cementing it into position in the ordinary way. After the cement has crystallized, a portion is removed at the cutting-edge and then thoroughly filled with gold as a protection to the cement. This operation is one of those frequently presented for restoration, and, when nicely done, is beautiful, durable, and always pleases the patient.

In Fig. 2 I show a bicuspid crown with a double porcelain face. I make this operation in cases where the patient objects to showing gold. The procedure is to make a band and bevel the surfaces alike to the gum-line, inside and out. I cut a hole in the cap and wax the post into position, and invest and solder before I grind the teeth, as it gives something to wax the teeth to, by letting the post extend quite a distance through the top of the gold cap. The teeth are selected and ground into position while the cap is on the root in the mouth. After removing the cap the teeth are backed up and waxed on, and the case is tried in the mouth, and, if found right, the case is invested, leaving the small surface where the two porcelain teeth are waxed together exposed. After the case is cleaned of wax it is heated to the proper point, and a small lump of solder (previously melted into a shot) is placed in position, and as it is brought to the melting-point it will, by its own weight, drop to its place between the porcelains, soldering them together and to the post. When this case is cemented into place on the root, it is ground so that the natural teeth strike first, sufficient to give them the blow before the new one strikes. If this be done in all cases of single crowns less trouble will be had from the breaking off of the porcelain teeth.

I have illustrated in Fig. 3 an instrument devised by me for root-trimming. The cut is twice the size at the cutting part, to have it well engraved. With this instrument the root can be readily shaped as desired, the guard keeping it from reaching or injuring the gum tissues.

Fig. 4 illustrates a helper, as I term it. I use this instrument for trimming roots, cleaning roots, and also for removing bands while fitting them to roots. It is indispensable in crown-work. In separating teeth I use paper disks, as they are flexible and cut just where you want them to. If I have no room to begin, I place a piece of cotton between them for one night, and plenty of room is obtained. I use a disk-carrier, which is illustrated in Fig 5. The time used in changing the disk is reduced to the minimum; the double-surface disk and the single-surface disk are used with this holder, and if I have a case which requires the use of twenty disks in separating or polishing, the whole time would not exceed one minute in changing. In working at crown- and bridge-work the greatest care should be taken to avoid the escape of gas, after laying the blow-pipe down.

I had been suffering from frequent headaches, and found the cause of it was faulty mechanism in my blow-pipe where the rubber tube was used as a means of regulating the flow of gas while the blow-pipe was in use. I discarded all the old devices, and had my mechanic make a pipe with a stop-cock in the shell, so that when I am through I can at once stop all leakage of gas, and I

at once recovered from my headache and have not been troubled since. Any mechanic can do this for you. I have illustrated this device in Fig. 6.

(To be continued.)

## Figures and Tables

**Fig. 1. f1:**
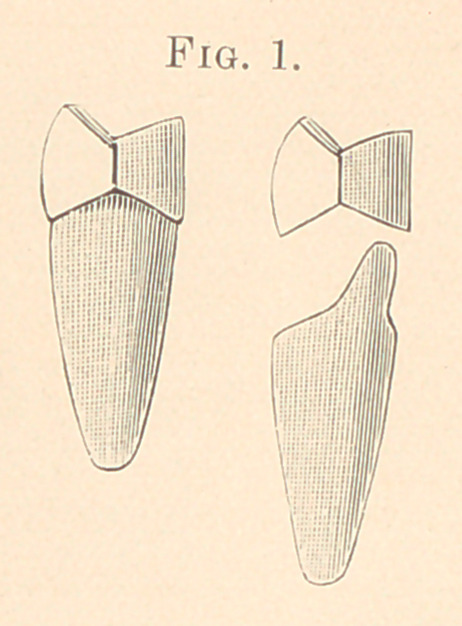


**Fig. 2. f2:**
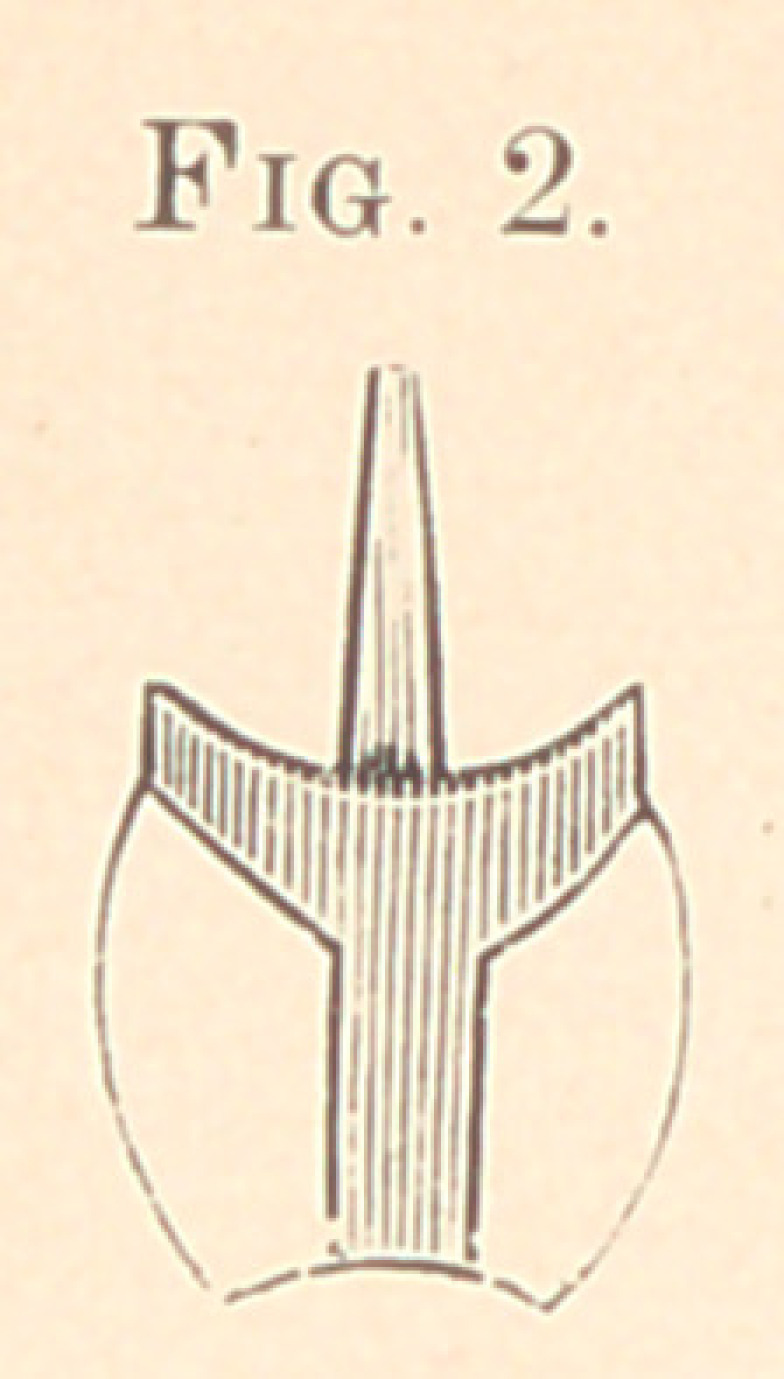


**Fig. 3. f3:**
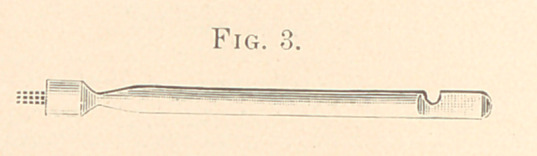


**Fig. 4. f4:**
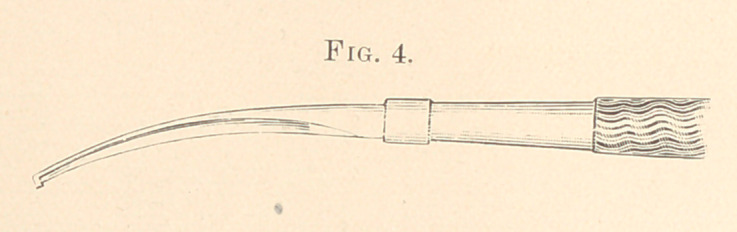


**Fig. 5. f5:**
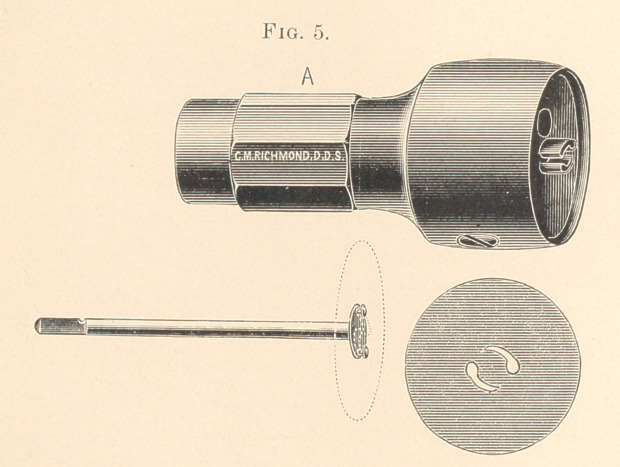


**Fig. 6. f6:**